# Scabies and Pediculosis in Penitentiary Institutions in Poland—A Study of Ectoparasitoses in Confinement Conditions

**DOI:** 10.3390/ijerph17176086

**Published:** 2020-08-21

**Authors:** Katarzyna Bartosik, Andrzej Tytuła, Zbigniew Zając, Weronika Buczek, Anita Jasztal-Kniażuk, Paweł Szczepan Błaszkiewicz, Adam Borzęcki

**Affiliations:** 1 Chair and Department of Biology and Parasitology, Medical University of Lublin, Radziwiłłowska 11 St., 20-080 Lublin, Poland; zbigniew.zajac@umlub.pl (Z.Z.); wera1301@gmail.com (W.B.); blaszkiewicz_pawel@interia.pl (P.S.B.); 2 Regional Chamber of Nurses and Midwives in Lublin, 20-072 Lublin, Poland; naszglos@o2.pl (A.T.); ajasztal@op.pl (A.J.-K.); 3 Med-Laser Non-Public Health Care Centre, 20-406 Lublin, Poland; laserlub@poczta.onet.pl

**Keywords:** *Sarcoptes scabiei* var. *hominis*, scabies, *Pediculus humanus*, pediculosis, neglected parasitic diseases, prisoner’s health, social stigma

## Abstract

Background: Scabies (caused by *Sarcoptes scabiei* var. *hominis*) and pediculosis (caused by *Pediculus humanus*) are infectious diseases common in educational institutions and long-term care centres. The aim of the study was to assess the scale of the phenomenon in confinement conditions favouring the spread of these parasitoses. Methods: Data on the prevalence of scabies (2001–2015) and pediculosis (2008–2015) in Polish prisoners were provided by the Central Board of Prison Service. The information for the period between 2010 and 2015 in the Lublin Province was obtained from the District Inspectorate of the Prison Service in Lublin. Correlations between the prevalence of scabies and pediculosis and the number of prisoners were analysed, as well as correlations between the number of passes granted to prisoners and the prevalence of scabies and pediculosis in incarcerated individuals. Results: The prevalence of scabies and pediculosis in Polish prisoners has been estimated at 2.3% and 1.9%, respectively. Conclusions: Pediculosis and scabies are still current issues in Polish prisons. Convicts returning from passes and new prisoners should be carefully examined and monitored for the presence of *S. scabiei* var. *hominis* and *P. humanus* invasion. Education of prisoners could be a promising tool in prevention of scabies and pediculosis in correctional settings.

## 1. Introduction

**Figure 1:**
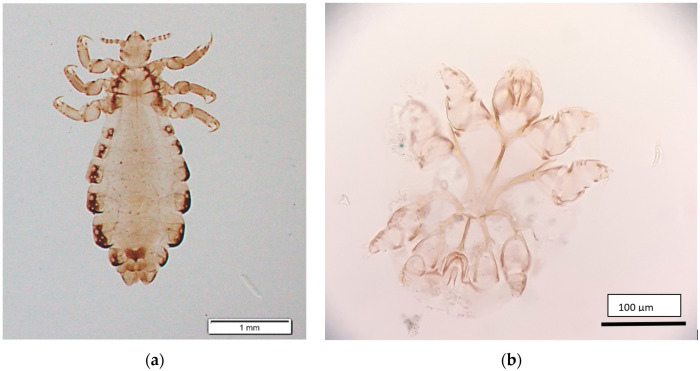
*Pediculus humanus* (**a**) and *Sarcoptes scabiei* var. *hominis* (**b**) (photographs by K. Bartosik).

Scabies and pediculosis are parasitic dermatoses caused by mites (*Sarcoptes scabiei* var. *hominis*) and insects (*Pediculus humanus*), respectively (Figure 1). These parasitic infections are some of the major public health problems not only in low and middle-income countries but also in highly developed ones [1,2,3,4,5,6]. The risk of parasite transmission is especially high in non-medical public and private facilities (e.g., long-term care facilities, hospitals, adult day care centres, schools, military facilities, and prisons). These facilities provide a special environment for parasite transmission, fostering frequent, close, and often intimate human-to-human contacts between residents. Factors that promote person-to-person transmission include close living quarters; a poor level of self-hygiene; sharing of fomites, e.g., combs and head gear; and sometimes a depressed mental state of residents [7]. High patient-to-staff ratios, high staff turnover, and inadequate implementation of infection control policies are challenges to the control of infections in non-medical public and private facilities [8,9]. In some countries (including Poland), reporting of scabies or pediculosis infection to local sanitary-epidemiological stations is not mandatory according to the law. Thus, the real scale of these parasitic infections is hard to assess. Previous studies estimated the prevalence of head lice around the globe in a range of 0.7–61.4% [10]. The overall worldwide prevalence of *S. scabiei* var. *hominis* infection ranges from 0.2% to 71.4% [11]. In highly developed countries, scabies infection occurs mainly in institutional settings such as hospitals, nursing houses, and prisons [8,12]. It is believed that confinement conditions favour the spread of ectoparasites, which necessitates rigorous hygienic control in penitentiary institutions. The aim of this study was to assess the prevalence of *P. humanus* and *S. scabiei* var. *hominis* infestation in confinement conditions under constant medical supervision in a period when reporting these parasitoses was no longer mandatory.

## 2. Materials and Methods

### 2.1. Data Collection

**Figure 2:**
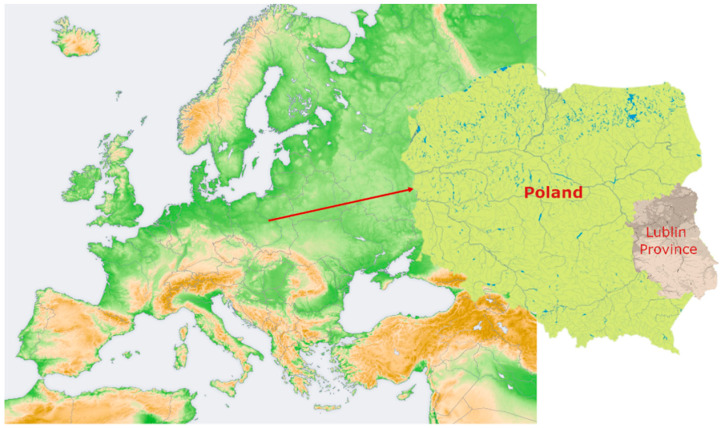
Study area at the national (Poland) and regional (Lublin Province) level, based on Wikimedia with our modifications.

The analysis of the prevalence of scabies and pediculosis in individuals residing in confinement conditions in Poland was based on epidemiological data collected in prisons both at the national and regional level (Figure 2). In these institutions, the ectoparasitoses are registered in compliance with internal procedures despite the lack of a statutory obligation to report their occurrence. Data on the prevalence of scabies (2001–2015) and pediculosis (2008–2015) in Polish prisoners were provided by the Central Board of Prison Service, while the information for the period between 2010 and 2015 in the Lublin Province was obtained from the District Inspectorate of the Prison Service in Lublin. The information on the number of passes granted to prisoners in 2001–2015 was taken from the Annual Statistical Information published online by the Ministry of Justice and the Central Board of the Prison Service. The constant monitoring of the statistics of pediculosis and scabies in penitentiary institutions imposed by the internal regulations of the prison service provides reliable information on the prevalence of these parasitoses in prisoners.

The data sets prepared by prison staff do not differentiate between pediculosis capitis and pediculosis corporis and include both invasions caused by *Pediculus humanus capitis* and *Pediculus humanus corporis*. Since our study was retrospective and non-invasive and all data were anonymous, no Ethics Committee approval was required.

### 2.2. Statistical Analysis

Analysis of the data was performed using statistical package PQStat ver. 1.4.2.324 (PQStat Software, Poznań, Poland). The correlation between the number of imprisoned individuals and scabies and pediculosis cases in the study period was analysed by estimation of Spearman’s rank correlation coefficient (r_s_). Probability was considered significant at *p* < 0.05 and highly significant at *p* < 0.01. At the national level, the correlation between the number of periodic passes granted to prisoners and the number of reported cases of scabies and pediculosis among prisoners was examined using the Pearson correlation coefficient.

## 3. Results

As demonstrated by data from 2001–2015, the average annual number of scabies cases in the study period was 1930 (Table 1).

**Figure 3:**
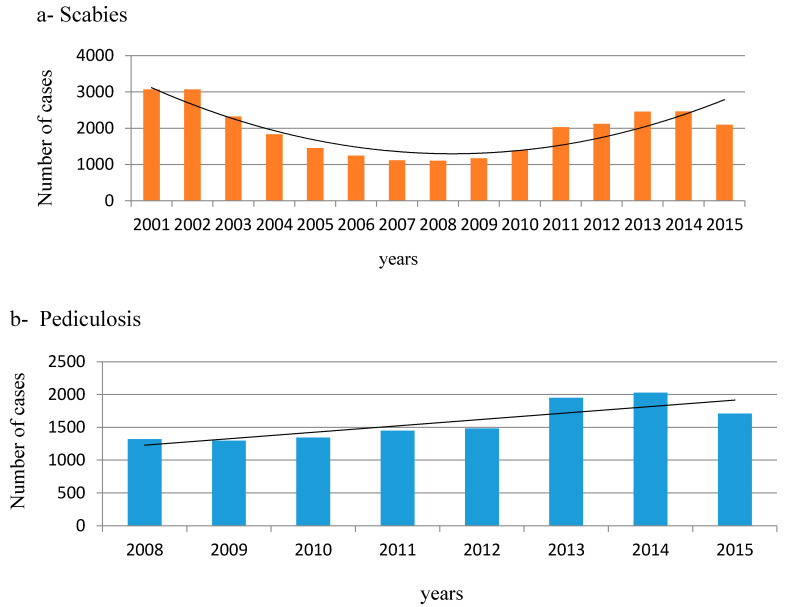
Number of cases of scabies and pediculosis with trend lines in Polish prisoners: (**a**) scabies in 2001–2015, (**b**) pediculosis in 2008–2015 (source: annual information, Central Board of Prison Service).

As shown by the trend line, the lowest number of scabies cases was recorded in 2007–2008, i.e., 1115 and 1103 cases, and the highest prevalence was noted in 2001–2002, i.e., 3072 and 3071 cases of the parasitosis (Figure 3a, Table 1). After 2003, the prevalence of infections gradually declined until 2010. Next, the number of reported scabies cases increased again. The graph shows a polynomial trend line. The median of the number of scabies cases in the study period was 2029 (SD = 627).

Based on the general data on the number of prisoners and the number of scabies infections, the prevalence (i.e., the extensiveness of infection) of scabies was calculated for the study years (Table 1). The highest prevalence of scabies was recorded in 2001, 2002, and 2014 (over 3% in relation to the total number of prisoners). The lowest percentage of infections was recorded in 2006–2010.

The higher number of prisoners in the analysed years (2001–2015) was not correlated to an increase in the prevalence of scabies: the greater the number of prisoners, the lower the number of infection cases (Rho Spearman = −0.718; *p* = 0.003).

The average number of cases of pediculosis among the prisoners in the analysed years was 1572. The highest and the lowest numbers of cases of the disease were recorded in 2014 and in 2009. From 2009, there was an increasing trend in pediculosis prevalence among the prisoners, which persisted until 2014. Decreasing prevalence of pediculosis was only noted in 2015 (Figure 3b).

As indicated above, the highest number of pediculosis cases, i.e., 2029, was reported in 2014, whereas the lowest number, i.e., 1296 cases, was noted in 2009 (Figure 3b, Table 2). The median of the number of pediculosis cases was calculated at 1466 (SD = 289). Based on general data on the number of prisoners and the number of pediculosis infections, the prevalence of the parasitism among prisoners was calculated in the study years, as in the case of scabies infection (Table 2). The highest percentage of pediculosis was recorded in 2013 and 2015 (over 2% in each year). There was no correlation between the number of prisoners and the prevalence of *P. humanus* invasion (r_s_ = −0.667; p = 0.071). A negative correlation between the total number of passes granted to prisoners and the incidence of head lice (r_s_ = −0.6933) and scabies (r_s_ = −0.7822) was noted.

The scale of the problem was analysed for the Lublin region as well. The data from the Regional Inspectorate of the Prison Service in Lublin originated from 2010–2015. Based on these data, it was concluded that the problem of scabies and pediculosis in 2010–2015 afflicted less than 1% of the prisoners in our Province. Throughout the study period, the prevalence of scabies and pediculosis was within the range of 0.3–1.0% and 0.1–1.1%, respectively (Table 3).

## 4. Discussion

Scabies and pediculosis are parasitic diseases spreading easily in confinement conditions [8,9,12,13,14,15]. In 2008–2015 in Poland, there were on average 82,708 prisoners in penitentiary institutions; scabies and pediculosis were diagnosed in 2.3% and 1.9% of the total number of prisoners, respectively. This percentage was significantly lower in the prisoners in the Lublin Province, i.e., 0.8% and 0.6%, respectively. The problem of skin parasitoses afflicts primarily male prisoners, since males constitute a majority of convicts in Poland (ca. 96%). These are more often middle-aged men (30–39 years old) and, to a lesser extent, young people below 20 years of age. Due to the changes in legal regulations introduced in 2008 and abolishing the obligation to report pediculosis cases by general practitioners and health care centres to relevant sanitary supervision units, only data from research studies, mainly involving schoolchildren, can serve as a reference point.

The level of head lice infestation in prisoners noted in our study (1.9%) was comparable with the average level of infection in the school-aged population in south-eastern Poland, in 2009–2012 (2.01%) [16]. The incidence of *P. humanus* invasion in the general population noted for the last time in 2008 was 0.006% [17]. In our study, the prisoners exhibited a significantly higher percentage of *S. scabiei* var. *hominis* infections than the non-incarcerated population. In the analysed period, on average 2.3% of the Polish prisoners suffered from scabies, whereas the incidence of this disease in Poland noted in the general population in 2008 was 0.029% [17].

The higher prevalence of skin parasites, i.e., pediculosis and scabies, in confinement institutions may be a result of the persistent overcrowding of prisons found all around the globe, leading to an increase in the number of prisoners per cell. The minimum surface area per prisoner in Poland has been legally specified at 4 m^2^; however, it can be reduced to only 2 m^2^ for up to 90 days, while this value in most European Union countries is substantially higher: 9 m^2^ in Belgium and 10 m^2^ in the Netherlands and Spain [18]. Polish prisons mainly have multi-person (4–10 persons) cells, while single cells are intended for particularly dangerous prisoners or used for solitary confinement. Inmates can communicate with each other (e.g., in cells, at meals, on walks, and at work in the prison) and with prison Staff, medical personel, or visitors. An important issue in the functioning of prisons is to maintain discipline among prisoners. Therefore, it should be taken into account that intense pruritus associated with the above-mentioned dermatoses may lead to anxiety [19] and, combined with the accumulation of individuals in a small space, may promote aggressive behaviour.

Health care services provided to Polish prisoners are financed entirely from the state budget.

In accordance with the provisions in force in Poland, prisoners are subjected to preliminary, periodic, and control medical examinations documented in the convict’s health records [20] and to sanitary procedures. Initial medical examinations are carried out by a general practitioner within 3 working days after imprisonment, while diagnostic examinations are performed within 14 days. This is a key time for elimination of ectoparasites, except for cases where the infestation is oligosymptomatic and/or the invasion is characterised by low intensity.

The spread of parasitic diseases of the skin, especially in the specific confinement conditions, is promoted by limited access to hygienic procedures. Non-working prisoners are entitled to only one bath with hot water a week, while women are entitled to two baths a week [18]. The conditions in cells that do not have adequate ventilation promote the out-of-host survival of scabies and louse [7,21,22,23,24]. Korycińska et al. described a positive correlation between air humidity and scabies incidence [25]. It should be mentioned that, in addition to the procedure of preliminary medical examinations followed by periodic and control check-ups, Polish prisoners have the right to make appointments mainly with the general practitioner [20]. Additionally, they are obliged to report their own disease and that of another convict to the prison service, which as a rule substantially accelerates the implementation of therapy for scabies and pediculosis.

As shown by the few available studies on the occurrence of pediculosis and scabies in prison conditions, a considerably higher prevalence of the infections than in Poland was recorded in prisons in Iran (0.9–5.2% in the case of louse invasion, and 1.2–2.2% in the case of scabies) [26,27,28]. Results obtained by Kuruvila et al. [29] in a district prison in Mangalore, India revealed that 63.6% of incarcerated individuals suffered from dermatoses and 8.0% of those had scabies and 6.6% pediculosis corporis. The literature also describes cases of scabies epidemics among prisoners in countries with a humid tropical climate, e.g., Tanzania and India, promoting infections [30,31]. Scabies is also the most common disease among prisoners in Cameroon [32].

Pediculosis has been shown to occur more frequently in drug-addicted prisoners and those sharing beds and bed linen as well as prisoners that bathe once a week or less frequently [27,28]. A study conducted by Nazari and coworkers showed that the occurrence of pediculosis was not affected by the educational level of the convict [26]; however, the knowledge about the epidemiology of pediculosis that could help prevent it is not by definition implemented during standard general education. That is why it should be propagated as a part of health education among prisoners. Adequate sanitary conditions, access to running water, reduction of the number of prisoners per cell, and detailed medical examinations of prisoners followed by several-day long quarantine may be important factors limiting the occurrence of scabies and pediculosis in confinement conditions [26,27,28,33,34].

Despite the existing medical supervision, ectoparasitoses in penitentiary institutions are still a current issue. Although the prevalence of scabies and pediculosis noted in our study is not considered epidemic (less than 4.0% for each), there is always a chance for improvement. Although our analysis did not confirm that the number of passes granted to prisoners had a significant impact on the prevalence of scabies and pediculosis in prisoners, newly convicted prisoners or those returning from leaves may be a source of the diseases. Therefore, special attention should be given to them both when they return to the facility and for the duration of the incubation period for these ectoparasitoses, i.e., 4–6 weeks in the case of scabies and 6–14 days in the case of pediculosis [35].

Studies conducted by Nazari and Azizi indicated association of the prevalence rate of scabies with the educational level; in subjects with a higher educational level, the prevalence of *S. scabiei* var. *hominis* invasion was over three times lower than in the illiterate group (13.0% and 41.2%, respectively) [36]. Among Polish prisoners, less than 6.0% are involved in both in-prison and out-of-prison schools. Obligatory educational programs can prevent the stigmatisation of the infected and dispel the common myths related to these diseases [37]. Making prisoners aware that they have a significant impact on limiting the invasion of ectoparasites in their own environment through preventive behaviours is beneficial from the social point of view [38]. It also gives prisoners a possibility to have an influence on their own existence, the lack of which is very severe in conditions of limited freedom. The results of preliminary research on the effects of health education on prisoners seem to be promising [39,40,41,42]. The effectiveness of health-promoting actions in reducing head lice infestation was confirmed in school students, another group particularly vulnerable to lice invasion e.g., [43,44]. Moreover, direct examination of indoor dust could be useful to detect the presence of mites and insects in an indoor environment [45].

## 5. Conclusions

Monitoring prisoners’ health facilitates instantaneous response to emerging cases of scabies and pediculosis, thereby contributing to limitation of their prevalence in these facilities. However, despite constant medical supervision, these parasitoses still occur in confinement conditions. In order to minimise the problem, additional tools in the form of health education should be considered to address this major public health issue.

## Figures and Tables

**Table 1:** Prevalence of scabies in Polish prisoners in 2001–2015 in relation to the number of imprisoned individuals and the number of cases noted.

Years	Number of Prisoners ^1^	Scabies	
		Number of Cases	Prevalence
2001	78,716	3072	3.9%
2002	81,391	3071	3.8%
2003	81,321	2324	2.9%
2004	80,239	1833	2.3%
2005	82,761	1455	1.8%
2006	87,370	1245	1.4%
2007	89,995	1115	1.2%
2008	85,920	1103	1.3%
2009	85,384	1172	1.4%
2010	82,863	1387	1.7%
2011	82,558	2029	2.5%
2012	84,399	2121	2.5%
2013	83,898	2455	2.9%
2014	78,987	2465	3.1%
2015	74,814	2096	2.8%
		**Total 28,943**	M * 2.3%

^1^ In 2001–2015, foreigners constituted on average 0.96% (from 0.65% to 2.03%) of the inmates. * Mean.

**Table 2:** Prevalence of pediculosis in Polish prisoners in 2008–2015 in relation to the number of imprisoned individuals and the number of cases noted.

Years	Number of Prisoners ^1^	Pediculosis	
		Number of Cases	Prevalence
2008	85,920	1319	1.5%
2009	85,384	1296	1.5%
2010	82,863	1344	1.6%
2011	82,558	1448	1.8%
2012	84,399	1484	1.8%
2013	83,898	1949	2.3%
2014	78,987	2029	2.6%
2015	74,814	1710	2.3%
		**Total 12,579**	M * 1.9%

^1^ In 2008–2015, foreigners constituted on average 0.67% (from 0.65 to 0.72%) of inmates. * Mean.

**Table 3:** Prevalence of scabies and pediculosis in prisoners in relation to the number of imprisoned individuals and cases noted in Lublin Province (2010–2015).

Years	Number of Prisoners	Scabies		Pediculosis	
		Number of Cases	Prevalence	Number of Cases	Prevalence
2010	4301	12	0.3%	9	0.2%
2011	4261	39	0.9%	6	0.1%
2012	4569	46	1.0%	32	0.7%
2013	4397	41	0.9%	49	1.1%
2014	4336	33	0.8%	36	0.8%
2015	4001	31	0.8%	32	0.8%
		**Total 202**	M * 0.8%	**Total 164**	M * 0.6%
		r_s_ = 0.714; p = 0.111		r_s_ = 0.522; p = 0.288	

* Mean.
